# Real-World Treatment Outcomes in the First and Subsequent Coronavirus Disease 2019 (COVID-19) Hospital Clusters

**DOI:** 10.7759/cureus.78981

**Published:** 2025-02-14

**Authors:** Yoshinobu Ohsaki, Takaaki Sasaki, Yasuhiro Umekage, Hiraku Yanada, Mariko Ishikawa, Ryohei Yoshida

**Affiliations:** 1 Clinical Research Center, Keiyukai Yoshida Hospital, Asahikawa, JPN; 2 Division of Respiratory Medicine and Neurology, Department of Internal Medicine, Asahikawa Medical University Hospital, Asahikawa, JPN; 3 Department of Infection Control, Asahikawa Medical University Hospital, Asahikawa, JPN

**Keywords:** covid-19, hospital, infection control, real-world survey, survival rate

## Abstract

Objectives: We experienced 10 coronavirus disease 2019 (COVID-19) hospital clusters between November 2020 and September 2023 and retrospectively examined whether the introduction of hospital cluster countermeasures improved patient prognosis.

Methods: We compared the first hospital cluster, in which infection prevention measures were insufficient, vaccines were not introduced, and antiviral drugs were not available (Phase 1), and the second or subsequent hospital clusters, when the abovementioned measures were improved (Phase 2).

Results: In Phase 2, the number of COVID-19 patient deaths within 30 days, infection rate in patients who shared a room with an infected patient, and infection rate among medical workers were reduced. Survival rates within 30 days did not differ significantly between Phases 1 and 2. In Phase 2, the survival rate was higher in females than in males and in groups treated with ensitrelvir and molnupiravir than in those treated with remdesivir.

Conclusions: Countermeasures against hospital clusters require comprehensive measures, such as infection prevention, vaccination, rapid diagnosis, and antiviral drug administration. Antiviral drugs may shorten hospital clusters by rapidly suppressing severe acute respiratory syndrome coronavirus 2 (SARS-CoV-2) infection in patients.

## Introduction

The coronavirus disease 2019 (COVID-19), which emerged in Wuhan, China, in December 2019, rapidly spread worldwide [1-3]. The first COVID-19 case in Japan, confirmed on January 16, 2020, involved a man in his 30s who had been in Wuhan and had developed pneumonia. The first case in Hokkaido involved a Chinese woman in her 40s who arrived in Japan on January 21, 2020, developed a fever on January 26, and was diagnosed with COVID-19 on January 28. Thereafter, COVID-19 began spreading sporadically throughout the prefecture.

In November 2020, our hospital in Asahikawa City experienced its first COVID-19 cluster, defined by the presence of five or more patients. The outbreak was considered over if no new cases emerged after 14 days (observation period). Despite normal infection control measures, information on COVID-19 infection patterns, prevention, and treatment was limited at this time. The hospital had limited rooms for proper cohorting and faced shortages of face shields, N95 masks, infection prevention gowns, and gloves. Owing to the lack of an infectious disease ward, patients were initially transported to a tertiary hospital in Asahikawa City; however, this hospital reached its capacity, forcing us to treat them at our hospital. Medical staff had to care for patients under inadequate infection control measures, wearing only surgical masks, which led to the rapid expansion of hospital clusters and infections among staff. Providing medical care became challenging, prompting us to request government assistance. No effective drugs against severe acute respiratory syndrome coronavirus 2 (SARS-CoV-2) were available. Clinical trials were being conducted for favipiravir (Avigan®, Fujifilm, Tokyo, Japan) [4] and ciclesonide (Alvesco®, Teijin Pharma, Ltd., Tokyo, Japan) [5], with approval on an emergency basis by the Ministry of Health, Labour and Welfare of Japan [6]. These drugs were administered to patients who provided consent. Vaccines had not yet been developed at that time.

The second hospital cluster developed between February 28 and March 28, 2022. Eight more clusters followed by September 2023. During this time, the definition of a hospital cluster was updated to seven or more patients, with the observation period shortened to seven days. Protective equipment supplies and cohorting procedures improved significantly. A 16-bed infectious disease ward was opened in September 2022 for treating infected patients. However, it was discontinued in September 2023 after the guidelines were updated by the Ministry of Health, Labour and Welfare of Japan.

After the second cluster, favipiravir and ciclesonide were discontinued, with most patients receiving remdesivir [7,8] or molnupiravir [9,10]. Following the approval of ensitrelvir [11] in March 2023, it was also administered to the infected patients. Specifically, patients who could take oral medication, were not taking contraindicated drugs, and had preserved renal function were treated with ensitrelvir; patients who were not taking contraindicated drugs or had reduced renal function were treated with molnupiravir; and patients who could not take oral medication and had more severe renal dysfunction were treated with remdesivir. Both patients and medical staff had received multiple doses of anti-SARS-CoV-2 vaccines by this time.

This study was conducted to determine whether the implemented measures improved patient survival in the hospital cluster. Verifying the effectiveness of these measures will provide important information for managing various future outbreaks. By comparing the first hospital cluster that occurred when the hospital was unprepared with later clusters that occurred when the hospital was better prepared, we sought insights for improving preparations and combating hospital clusters in future pandemics.

## Materials and methods

Keiyukai Yoshida Hospital has six wards, which are categorized into four functional groups: a community comprehensive care ward (first and second floors), a general internal medicine ward (fourth and seventh floors), a disability ward (fifth and sixth floors), and a palliative care ward (fifth floor). It provides care for approximately 210 patients, many of whom are older individuals with multiple health problems and physical disabilities, including those with dementia, frailty, multiple cerebral infarctions, chronic aspiration, and chronic heart failure. Nursing staff often assist patients with tasks such as moving, bathing, eating, and toileting, which require close patient contact. The hospital's infection control team, consisting of a doctor, nurse, pharmacist, and laboratory technician, adheres to standards set by the Ministry of Health, Labour and Welfare of Japan.

Until January 2021, SARS-CoV-2 infection at Asahikawa City Public Health Center was confirmed by polymerase chain reaction testing, conducted once or twice a week, with results available after 24 hours. From January 2021, loop-mediated isothermal amplification [12] and antigen quantitative tests were used, and these were eventually replaced by antigen quantitative testing alone. The tests were conducted on hospitalized patients and medical staff experiencing COVID-19 symptoms, such as fever, sore throat, and cough. Roommates of infected patients were considered close contacts and managed through cohorting, with periodic checks for infection and SARS-CoV-2 detection.

The first hospital cluster at our institution occurred on November 6, 2020, and the second hospital cluster occurred on February 28, 2022. On August 24, 2023, the 10th hospital cluster was recognized. As mentioned above, the conditions of the first, second, and subsequent clusters differed notably. For the purpose of this study, we defined the first cluster as Phase 1 and the second and later clusters as Phase 2. In Phase 2, patients diagnosed with SARS-CoV-2 infection were immediately prescribed antiviral medication if indicated.

To determine changes between Phase 1 and Phase 2, hospital medical records were retrospectively reviewed to collect information on patient sex, age, date of COVID-19 onset, date of death (if applicable), whether death was due to COVID-19, administration of antiviral drugs, feeding status, and patient movement. The number of COVID-19 vaccine doses administered to the patients was recorded. Patients who shared rooms with infected individuals at the time of the cluster outbreak, but remained uninfected, were identified using the hospital's bed-control history. Health records were also reviewed to identify the medical staff infected with SARS-CoV-2 during each hospital cluster.

Statistical analysis

Indicators were compared between Phases 1 and 2 using Fisher's exact test. Factors related to survival were selected using the Cox proportional hazards model. Survival curves were constructed using the Kaplan-Meier method and were compared using the log-rank test. All statistical analyses were performed using the Mac Statistical Analysis Version 3.0 (Esumi Co., Tokyo, Japan). Statistical significance was set at p<0.05.

Ethical consideration

Approval to conduct the study was obtained from the Yoshida Hospital Ethics Committee (approval number: 20231108001) on November 9, 2023. Opt-out information was published on the Yoshida Hospital website before the start of the study in lieu of obtaining participant consent for data use.

## Results

Table 1 and Table 2 show the characteristics of the 10 hospital clusters that our hospital experienced from November 2020 to September 2023.

**Table 1:** Phase 1 analysis of the cluster at Yoshida Hospital ^a^Excluding the observation period from the cluster period, 14 days for PI and seven days for PII. ^b^Infected patients. ^c^Patients diagnosed per day. ^d^Patients in the same room who did not become infected. ^e^Infected medical staff.^ f^Medical staff diagnosed per day F: floor; PI: Phase 1; PII: Phase 2

Clusters		Duration	Days^a^	Ward	Infected patients^b^	Patients diagnosed/day^c^	Non-infected patients^d^	Infected med. staff^e^	Medical staff diagnosed/day^f^
Phase 1	1st	20/11/6-21/1/2	43	1/2-7F	136	3.16	24	78	1.81

**Table 2:** Phase 2 analysis of the cluster at Yoshida Hospital ^a^Excluding the observation period from the cluster period, 14 days for PI and seven days for PII. ^b^Infected patients. ^c^Patients diagnosed per day. ^d^Patients in the same room who did not become infected. ^e^Infected medical staff.^ f^Medical staff diagnosed per day F: floor; PI: Phase 1; PII: Phase 2; P: palliative care unit

Clusters		Duration	Days^a^	Ward	Infected patients^b^	Patients diagnosed/day^c^	Non-infected patients^d^	Infected med. staff^e^	Medical staff diagnosed/day^f^
Phase 2	2nd	22/2/28-3/28	21	4F	8	0.38	3	2	0.10
	3rd	22/8/16-8/31	8	P	5	0.63	0	3	0.38
	4th	22/9/25-10/8	6	4F	8	1.33	3	0	0
	5th	22/10/21-11/16	19	1/2,7F	36	1.89	21	5	0.26
	6th	22/11/22-12/20	21	1/2,4,6F,P	35	1.67	18	17	0.81
	7th	23/1/11-2/1	14	1/2,7F	17	1.21	27	3	0.21
	8th	23/4/25-5/23	21	1/2,7F	20	0.95	10	3	0.14
	9th	23/6/3-6/19	9	4F	9	1.00	9	3	0.33
	10th	23/8/24-9/19	19	4,7F	22	1.16	17	11	0.58
Total			-	-	160	-	108	47	-
Average			15.3	-	19.2	1.14	-	-	0.31

In Phase 1, the indirect period was set at 14 days without any new COVID-19 cases. In Phase 2, this was shortened to seven days. Therefore, the number of days in a cluster was calculated by subtracting 14 days from the duration of the cluster in Phase 1 and seven days in Phase 2. Thus, the average number of days in a hospital cluster was 43 in Phase 1 and 15.3 in Phase 2, ranging from eight to 21 days. In Phase 1, 136 patients were infected, with an average daily infection rate of 3.16. On November 6, 2020, when the first hospital cluster occurred, the total number of hospitalized patients was 216. Among patients sharing a room with infected patients, 24 did not contract COVID-19. Among the medical staff, 78 were infected, with an average daily infection rate of 1.81. In Phase 2, 160 patients were diagnosed with COVID-19, with an average of 1.14 cases per day, and 108 patients in the same room as the infected patients remained uninfected. In total, 47 medical staff members became infected, with an average daily rate of 0.31.

In Phase 1, 136 patients (57 men, 79 women) were infected, with an average(±standard deviation) age of 83.2±10.0 years. In Phase 2, 160 patients (72 men, 88 women) were infected, with an average age of 83.2±8.9 years. No significant bias was observed in sex or age distribution (Table 3). 

**Table 3:** Analysis of the cluster at Yoshida Hospital ^a^Men/women. ^b^Average±SD. ^c^Death within 30 days. ^d^Death within 30 days due to COVID-19 F/C/+: treated by favipiravir or ciclesonide or with both; M/R/E: treated by molnupiravir or remdesivir or ensitrelvir; *: two cases with sotrovimab; **: one case with molnupiravir+sotrovimab; COVID-19: coronavirus disease 2019

Clusters		Pts	m/w^a^	Age^b^	Treatment received		<30 days^c^	<30 by COV^d^
					F/C/+	M/R/E		
Phase 1	1st	136	57/79	83.2±10.0	23/16/13	-	39/28.7%	30/22.1%
Total		136	57/79	83.2±10.0	23/16/13	-	39/28.7%	30/22.1%
Phase 2	2nd	8	1/7	89.7±4.9	-	5/0/0	0	0
	3rd	5	4/1	82.7±9.8	-	3/0/0	4	2
	4th	8	6/2	87.8±6.8	-	4/4/0	3	2
	5th	36	16/20	82.1±10.4	-	25/8/0*	6	4
	6th	35	14/21	81.0±8.3	-	14/19/0**	15	9
	7th	17	9/8	85.0±6.9	-	6/10/1	4	2
	8th	20	8/12	82.2±8.8	-	3/7/9	2	0
	9th	9	7/2	86.8±9.6	-	2/2/3	2	1
	10th	22	7/15	84.7±7.8	-	1/14/6	0	0
Total		160	72/88	83.2±8.9	-	63/64/19	36/22.5%	20/12.5%

The number of patients treated in each phase is presented in Table 3. The observation period for the purpose of this study was set to 30 days, and those whose medical records stated the cause of death as COVID-19 were considered to have died due to COVID-19. Thirty-nine patients in Phase 1 died within 30 days of diagnosis. Among them, 30 deaths were attributed to COVID-19. The 30-day case fatality rate of COVID-19 in Phase 1 was 30/136 (22.1%). In Phase 2, 36 infected patients died within 30 days of diagnosis. Of these, 20 deaths were attributed to COVID-19, resulting in a 30-day case fatality rate of 20/160 (12.5%).

Table 4 shows the vaccination status in Phase 2.

**Table 4:** Number of vaccinations in Phase 2

Cluster	No.	Pts	0	1	2	3	4	5	6	Unknown
Phase 2	2nd	8	-	-	5	-	-	-	-	3
	3rd	5	1	-	1	3	-	-	-	-
	4th	8	2	-	1	3	1	-	-	1
	5th	36	3	1	2	6	16	-	-	8
	6th	35	1	-	2	7	16	1	-	8
	7th	17	-	-	-	4	6	4	-	3
	8th	20	3	-	-	3	4	3	-	7
	9th	9	-	-	-	-	1	5	1	2
	10th	22	-	-	-	1	1	6	4	10
Total		160	10	1	11	27	45	19	5	42

In total, 10 of the 160 infected patients had not been vaccinated, and the number of vaccinations increased in later clusters. Vaccination history could not be confirmed for 42 patients (Table 4).

The results of Fisher's exact test (Table 5 and Table 6) showed that, in Phase 2, compared with Phase 1, fewer patients died from COVID-19 within 30 days (p<0.05), more patients who shared rooms with infected patients did not contract the infection (p<0.01), and fewer infections occurred among medical staff (p<0.01).

**Table 5:** Comparison between Phase 1 and Phase 2: deaths from COVID-19 within 30 days Indicators were compared between Phases 1 and 2 using Fisher's exact test, and the p-values were calculated accordingly COVID-19: coronavirus disease 2019

Phase	Deaths	Survivors	Total	Fatality (%)	P-value
Phase 1	30	106	136	22.1	p=0.0305
Phase 2	20	140	160	12.5	
Total	50	246	296	16.9	

**Table 6:** Comparison between Phase 1 and Phase 2: infections among medical staff Indicators were compared between Phases 1 and 2 using Fisher's exact test, and the p-values were calculated accordingly

Phase	Infected patients	Infected medical staff	Total	P-value
Phase 1	136	78	214	p=0.0027
Phase 2	160	47	207	
Total	296	125	421	

Factors associated with the risk of death were analyzed using a Cox proportional hazards model. For this analysis, the patients' mode of nutrition intake was classified into oral intake, feeding tube intake, and infusion; we compared oral intake with feeding tube intake plus infusion. For patient transportation methods, comparisons were made between walking, wheelchair transportation, and stretcher transportation. The average age of patients with COVID-19 in both phases was 83 years; therefore, the analysis was stratified into those aged 83 years or younger and those aged over 84 years. 

In Phase 1, sex, age, mode of nutrition intake, and transportation method were not found to be significant risk factors. The risk of death was slightly higher in patients undergoing hemodialysis than in those not undergoing hemodialysis in Phase 1; however, the difference was not statistically significant (hazard ratio (HR) 0.45; 95% confidence interval (CI) 0.20-1.05; p=0.065) (data was not shown).

In Phase 2, sex was a significant influencing factor, with women having a higher survival rate than men (HR 0.28: 95% CI 0.14-0.58; p<0.01). Hemodialysis, which was a marginal risk factor in Phase 1, was not a significant risk factor for 30-day mortality in Phase 2 (HR 5.30; 95% CI 0.71-39.5; p>0.05).

**Figure 1:**
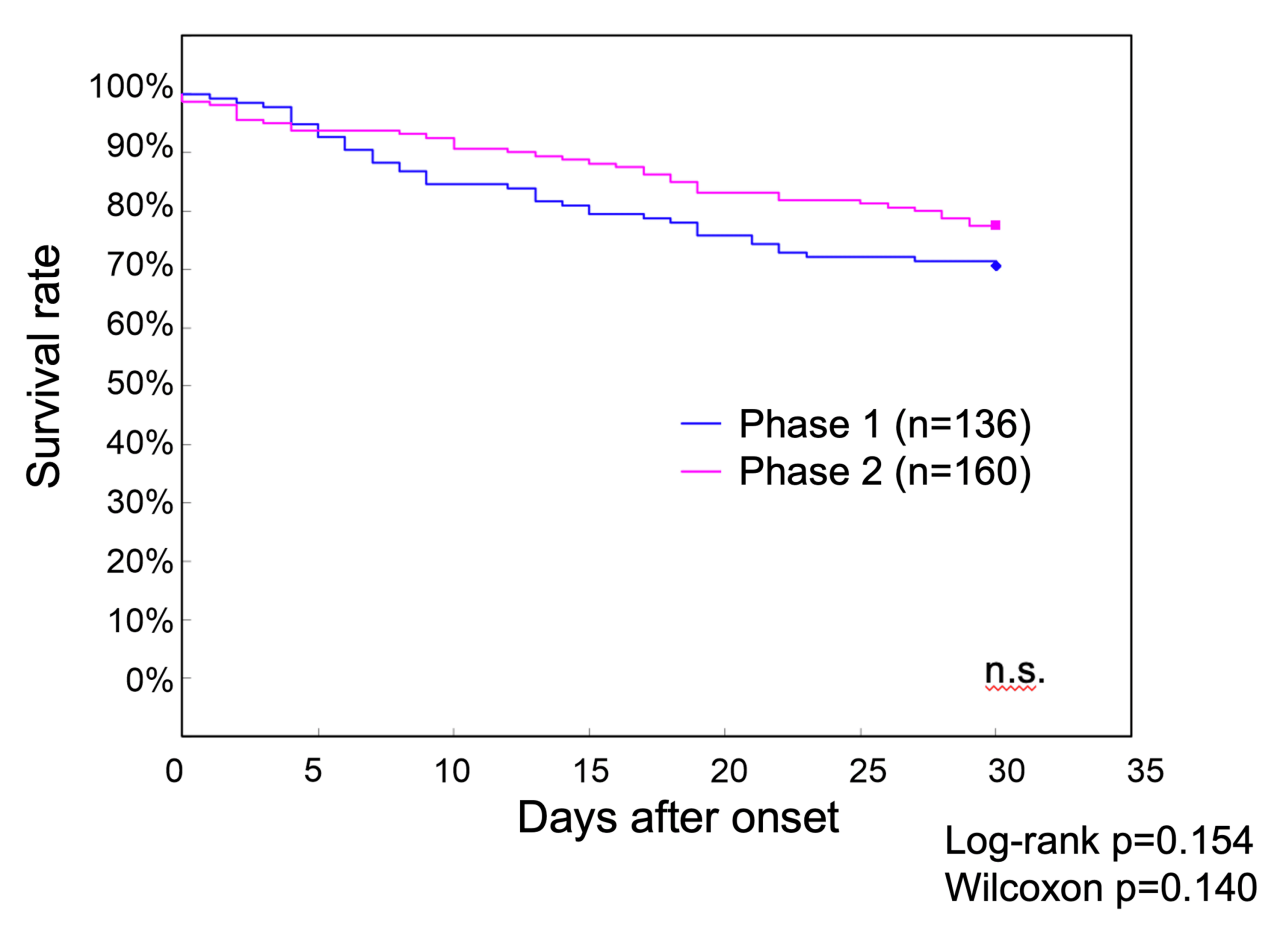
Survival curves of patients with COVID-19 up to 30 days after onset during Phase 1 and Phase 2 at our hospital The 30-day survival rate was 70.6% in Phase 1 and 77.5% in Phase 2, without a statistically significant difference COVID-19: coronavirus disease 2019; n.s.: nonsignificant

Survival curve analysis comparing the 136 Phase 1 patients and 160 Phase 2 patients showed 30-day survival rates of 70.6% in Phase 1 and 77.5% in Phase 2, although the difference was not statistically significant (Figure 1). 

**Figure 2:**
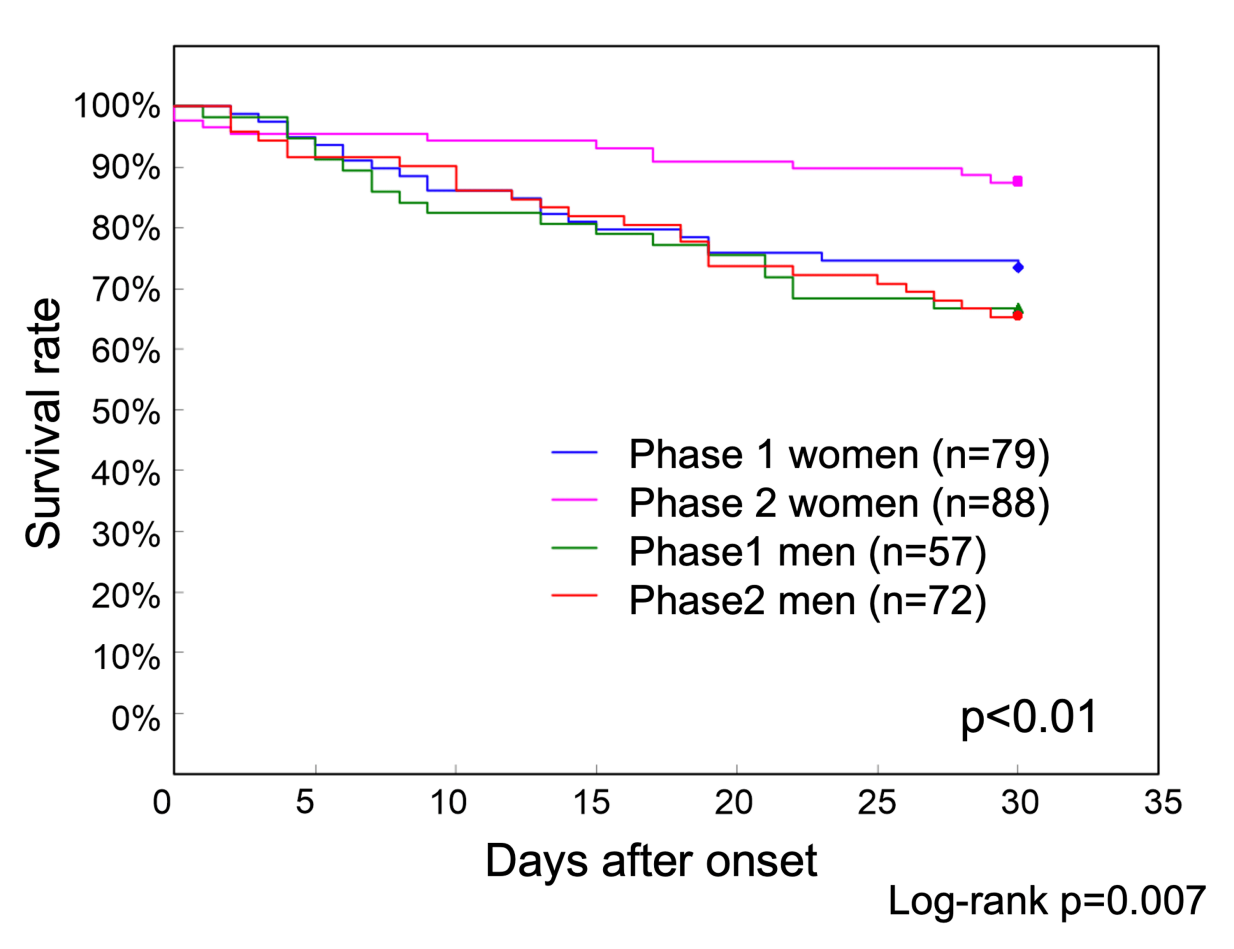
Phase 1 and Phase 2 survival curves by sex up to 30 days after onset The 30-day survival rates were 66.7% for men and 73.4% for women in Phase 1 and 65.3% for men and 87.5% for women in Phase 2. The survival rate of women in Phase 2 was significantly higher than that of men in Phase 2 and that of both men and women in Phase 1 (p<0.01)

Because the risk of death was significantly lower in women in the risk factor analysis of Phase 2, survival curves for Phases 1 and 2 were also examined by sex (Figure 2).

The 30-day survival rates were 66.7% (38/57) for men and 73.4% (58/79) for women in Phase 1 and 65.3% (47/72) for men and 87.5% (77/88) for women in Phase 2. The survival rate of women in Phase 2 was significantly higher than that of men in Phase 2, as well as that of both men and women in Phase 1.

**Figure 3:**
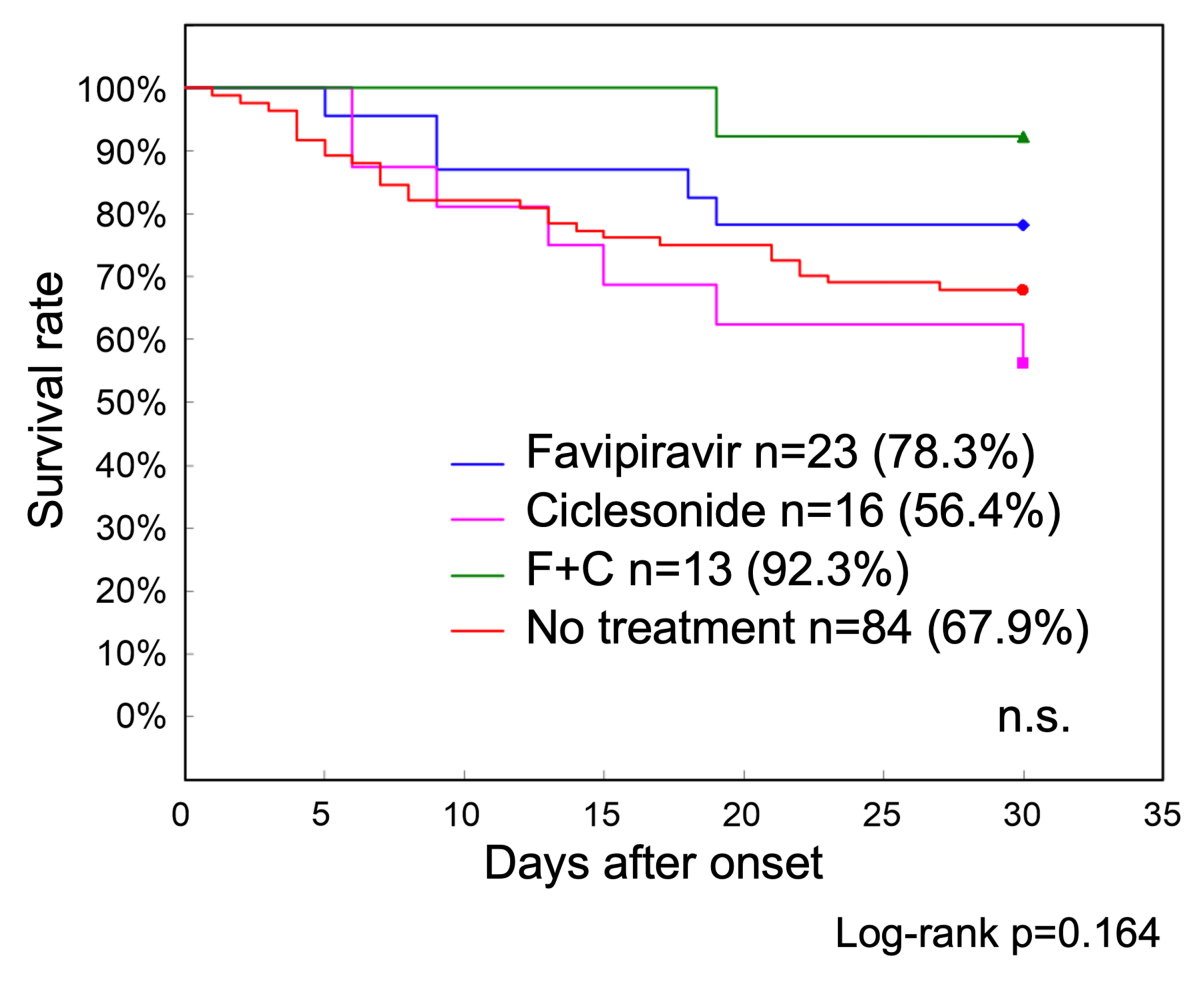
Phase 1 treatment-specific survival curves up to 30 days after onset The 30-day survival rate was 67.9% in 84 patients with COVID-19 who did not receive antiviral drug treatment, as compared with 78.3% in 23 patients who received favipiravir, 56.4% in 16 patients who received ciclesonide, and 92.3% in 13 patients who used both these drugs. Although the survival rate was slightly better in the combination treatment group, no statistically significant differences were observed COVID-19: coronavirus disease 2019: n.s.: nonsignificant

Of the 136 patients with COVID-19 in Phase 1, 52 were treated with favipiravir, ciclesonide, or a combination of the two drugs (Table 3). Survival curve analysis showed that the 30-day survival rate was 67.9% in 84 patients who did not receive these treatments, compared with 78.3% in 23 patients who received favipiravir, 56.4% in 16 patients who received ciclesonide, and 92.3% in 13 patients who received both drugs, indicating that the survival rate was slightly better in the combination group; however, no statistically significant differences were observed (Figure 3).

**Figure 4:**
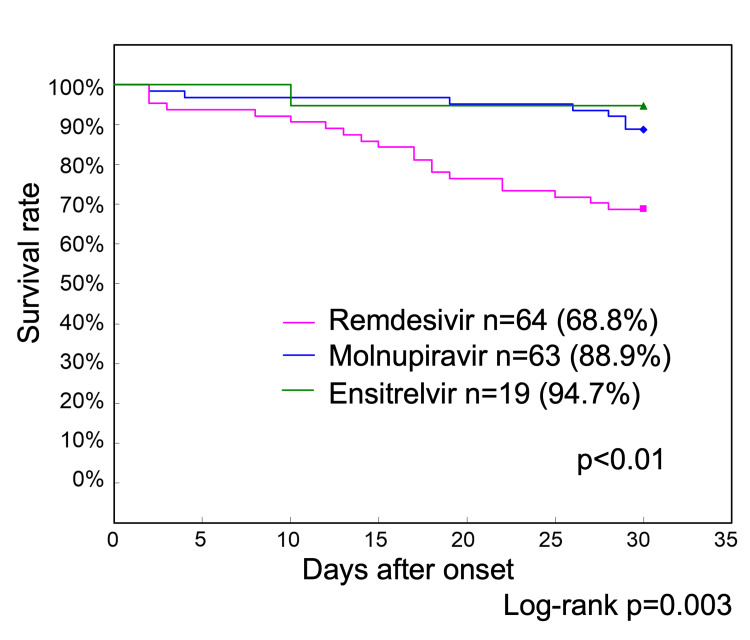
Phase 2 treatment-specific survival curves up to 30 days after onset The 30-day survival rate of 19 patients with COVID-19 who received ensitrelvir was 94.7%, and that of 63 patients who received molnupiravir was 88.9%. In contrast, the survival rate of 64 patients who received remdesivir was 68.8%, which was significantly lower than that of those who received ensitrelvir and molnupiravir (p<0.01) COVID-19: coronavirus disease 2019

In Phase 2, 63 patients were treated with molnupiravir, 64 with remdesivir, and 19 with ensitrelvir. Additionally, two patients received sotrovimab, and one received a combination of molnupiravir and sotrovimab. The remaining 11 patients were ineligible for treatment because of poor general health. Survival curves after treatment were analyzed, excluding the two patients who received sotrovimab, one who received a combination of molnupiravir and sotrovimab, and 11 who did not receive treatment. The 30-day survival rate of the 19 patients who received ensitrelvir was 94.7% and that of the 63 patients who received molnupiravir was 88.9% (Figure 4).

In contrast, the survival rate of the 64 patients who received remdesivir was 68.8%, which was significantly lower than that of the ensitrelvir and molnupiravir groups (p<0.01).

We also compared the effect of the above treatments for men and women separately in Phase 2 (data not shown). Remdesivir was administered to 34 women and 30 men, with no significant sex bias. The mean age of patients receiving remdesivir was 86.4±7.9 years (range: 69-103 years; median: 87 years), which was higher than that of the patients with COVID-19 overall. Molnupiravir and remdesivir were administered almost equally to both men and women, while ensitrelvir was administered slightly more often to women (13 women and six men). However, there was no significant bias in the sex ratio for the overall medication status.

**Figure 5:**
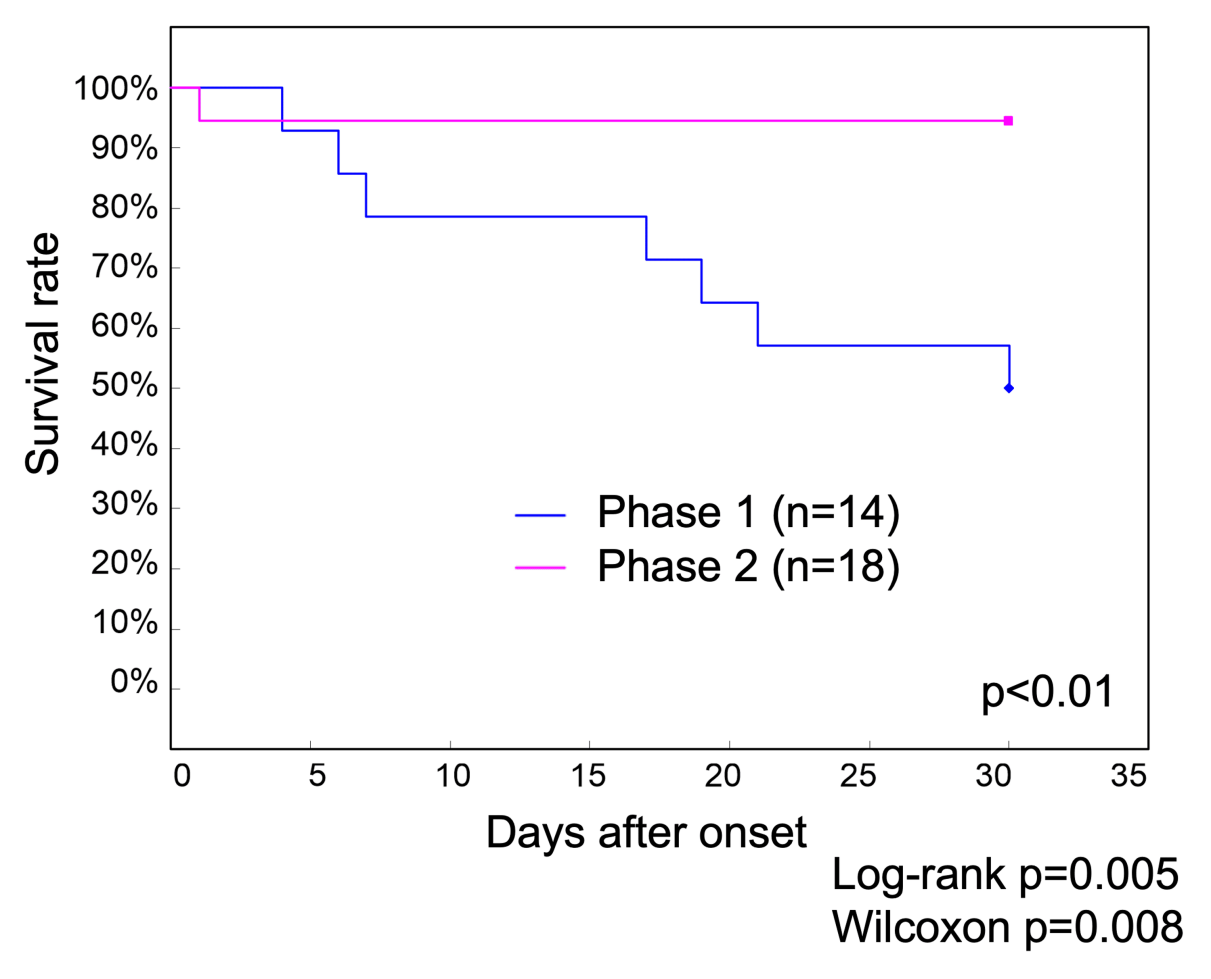
Survival curves for patients with COVID-19 on hemodialysis in Phase 1 and Phase 2 In Phase 1, 14 patients were on hemodialysis, compared to 18 in Phase 2. Molnupiravir was administered to 16 of the 18 patients, and remdesivir was administered to one patient. In one patient, no anti-SARS-CoV-2 drug was administered, and the patient died on the first day of COVID-19 diagnosis COVID-19: coronavirus disease 2019; SARS-CoV-2: severe acute respiratory syndrome coronavirus 2

As hemodialysis was a marginal risk factor in Phase 1, although not in Phase 2, we examined the survival curves of patients undergoing hemodialysis specifically in both Phases 1 and 2 (Figure 5).

In Phase 1, 14 infected patients were on hemodialysis (seven men and seven women), of whom seven died within 30 days (four men and three women). In Phase 2, 18 infected patients were on hemodialysis (10 men and eight women), and one woman died on the first day after COVID-19 diagnosis. Molnupiravir was administered to 16 of the 18 patients on hemodialysis in Phase 2, while remdesivir was administered to one patient. The female patient who died on day 1 after COVID-19 diagnosis could not receive any treatment.

## Discussion

Our study showed that compared with the first hospital cluster of COVID-19, subsequent clusters (second to 10th) had fewer COVID-19-related deaths, fewer infected roommates, and lower infection rates among medical staff. The Phase 2 clusters were shorter and involved fewer patients than the Phase 1 clusters. The first cluster occurred when sufficient measures and space were unavailable for patient isolation. Patients sharing a room with an infected patient were managed there and were thus high-density contacts, which led to a chain of transmission. Moreover, many medical staff members were infected, reducing the number of staff available for patient care. When a hospital cluster occurred in November 2020, the deployment of infection control personnel, replenishment of prevention equipment, and deployment of medical staff through the government aided in ending the hospital cluster. As it is unrealistic for hospitals to stockpile infection prevention equipment or flexibly allocate medical staff in preparation for an outbreak of unknown infectious diseases, our results suggest that hospitals should proactively establish close coordination with local hospital networks and government agencies involved in medical care.

In Phase 2, the hospital cluster size decreased compared to that in Phase 1, likely due to the sophistication of infection control measures and the widespread availability of vaccines; moreover, the introduction of therapeutic drugs may have played a major role. Another factor was that the time to diagnosis was shortened by switching from polymerase chain reaction testing at public institutions to rapid testing at our hospital. This allowed infected patients to be isolated quickly and antiviral drugs to be administered. The number of non-infected patients who were in the same room as an infected patient compared to the total number of infected patients significantly increased in Phase 2. This is likely due to the effects of the vaccine and treatment, in addition to the fact that the diagnosis of infected patients was made earlier, making it possible to rapidly isolate them. It is also possible that SARS-CoV-2 mutations had altered the pathogenesis of COVID-19. However, our study could not determine which of these factors was most associated with a reduced hospital cluster size in Phase 2.

The average age of the patients in Phases 1 and 2 was 83 years. Reportedly, the Omicron strain caused an increase in the deaths of older people due to intercurrent illnesses and worsening complications triggered by viral infections [13]. For older adults, determining the cause of death is difficult due to their physical condition. In Japan, the standard for reporting deaths due to COVID-19 was "death during the treatment period." In this study, considering the difficulty of determining the cause of death in older people, we examined all deaths that occurred within 30 days as well as those defined as COVID-19-related deaths that occurred within 30 days after diagnosis.

In Phase 1, the medical system collapsed due to successive infections of medical staff, which hampered the provision of adequate medical care. In Phase 2, although infections occurred among medical workers, the infection rate remained low. This is thought to be due to improvements in hospital management, such as the appropriate use of protective equipment and ingenuity in cohorting. The lower number of infections among the medical staff may have contributed to the decreased number of COVID-19-related deaths within 30 days in Phase 2. However, as it has been reported that medical staff may become infected even if protective equipment is used appropriately [14], more attention must be paid to the management of the environment in terms of infection prevention.

The survival rate was better in women in Phase 2 of our study. The use of the antiviral drugs molnupiravir and remdesivir did not differ significantly between men and women, whereas ensitrelvir was used slightly more frequently by women. In Japan, women have a longer life expectancy than men; therefore, the survival rate of older women is higher than that of men. In addition, some analyses of COVID-19 mortality rates have reported higher survival rates in women than in men [15,16]. Our study was unable to clarify why the survival rate was higher in women in Phase 2.

Favipiravir is an RNA-dependent RNA polymerase inhibitor developed for the treatment of influenza and has been approved in Japan for the treatment of influenza and severe fever with thrombocytopenia syndrome. A clinical observational study of favipiravir as a treatment for COVID-19 ended on December 28, 2021. Prior to this observational study, clinical trials suggested the efficacy of favipiravir [17]. However, on October 14, 2022, Fujifilm announced the discontinuation of favipiravir as a treatment for COVID-19 [18]. The inhaled steroid ciclesonide had been approved in Japan as a treatment for bronchial asthma. Regarding ciclesonide, the National Center for Global Health and Medicine concluded on December 23, 2020, that the rate of pneumonia exacerbation was significantly higher in the ciclesonide inhalation group than in the symptomatic treatment group. In this retrospective study, we only briefly reported the results of 52 patients treated with favipiravir, ciclesonide, or both in combination in Phase 1.

The 30-day survival rate of patients treated with remdesivir was significantly lower than that of patients treated with ensitrelvir or molnupiravir in this study. The average age of patients who received remdesivir was higher than that of patients with COVID-19 overall. Patients who were administered remdesivir may have had worse systemic conditions than those administered ensitrelvir or molnupiravir. When examining the factors related to survival risk, neither the mode of nutrition intake nor the method of transportation was a significant factor. We consider that the lower survival rate of the remdesivir group versus the other treatment groups may have reflected differences in diet, patient transportation, renal dysfunction, and coexisting physical disabilities. Although our study was a small-scale study conducted at a single facility, it showed that antiviral drugs, particularly molnupiravir, may be effective in patients undergoing hemodialysis. Molnupiravir is metabolized in the liver, whereas remdesivir can be used regardless of the degree of renal dysfunction; both drugs are currently recommended for use in patients undergoing hemodialysis [19,20]. Anti-SARS-CoV-2 drugs, including ensitrelvir, have been reported to reduce viral antigen expression [21]. Reducing viral shedding in patients with COVID-19 is expected to reduce the number of infections in hospitals. Administering anti-SARS-CoV-2 drugs early in the course of COVID-19 may control the transmission of the virus from infected patients and help to quickly end hospital clusters.

This study had some limitations. It was a small-scale, retrospective study conducted at a single facility. Large-scale comparative trials are typically needed to introduce new drugs into clinical practice. However, even with approved drugs, information on whether they can be applied to various clinical case scenarios is often insufficient. When used on a small scale, the efficacy of such drugs may be unclear. The significance of our findings is that we have demonstrated that a single measure is insufficient to fight hospital clusters. A comprehensive approach involving infection control measures, vaccinations, and antiviral drugs is essential.

## Conclusions

This study underscores the importance of a multifaceted approach in managing hospital clusters during the COVID-19 pandemic. The implementation of enhanced infection control measures, widespread vaccination, and timely administration of antiviral medications significantly reduced the rates of infection among patients and medical staff, shortened the duration of hospital clusters, and improved survival outcomes. In particular, molnupiravir demonstrated promising efficacy for patients undergoing hemodialysis, highlighting the potential benefits of tailored treatments for high-risk groups. These findings emphasize the necessity of proactive strategies, including early diagnosis and personalized therapeutic interventions, to mitigate the impact of infectious disease outbreaks and enhance hospital preparedness for future public health challenges.
